# Estimating ancestral proportions in a multi-ethnic US sample: implications for studies of admixed populations

**DOI:** 10.1186/1479-7364-6-2

**Published:** 2012-07-05

**Authors:** Orna Levran, Olaoluwakitan Awolesi, Pei-Hong Shen, Miriam Adelson, Mary Jeanne Kreek

**Affiliations:** 1 The Laboratory of the Biology of Addictive Diseases, The Rockefeller University, New York, NY, 10065, USA; 2 Laboratory of Neurogenetics, National Institute on Alcohol Abuse and Alcoholism, Bethesda, MD, 20892, USA; 3 The Dr. Miriam and Sheldon G. Adelson Clinic for Drug Abuse Treatment and Research, Las Vegas, NV, 89169, USA

**Keywords:** Ancestry informative markers, Hispanics, African Americans, Family history, Ancestry, Admixture

## Abstract

This study was designed to determine the ancestral composition of a multi-ethnic sample collected for studies of drug addictions in New York City and Las Vegas, and to examine the reliability of self-identified ethnicity and three-generation family history data. Ancestry biographical scores for seven clusters corresponding to world major geographical regions were obtained using STRUCTURE, based on genotypes of 168 ancestry informative markers (AIMs), for a sample of 1,291 African Americans (AA), European Americans (EA), and Hispanic Americans (HA) along with data from 1,051 HGDP-CEPH ‘diversity panel’ as a reference. Self-identified ethnicity and family history data, obtained in an interview, were accurate in identifying the individual major ancestry in the AA and the EA samples (approximately 99% and 95%, respectively) but were not useful for the HA sample and could not predict the extent of admixture in any group. The mean proportions of the combined clusters corresponding to European and Middle Eastern populations in the AA sample, revealed by AIMs analysis, were 0.13. The HA subjects, predominantly Puerto Ricans, showed a highly variable hybrid contribution pattern of clusters corresponding to Europe (0.27), Middle East (0.27), Africa (0.20), and Central Asia (0.14). The effect of admixture on allele frequencies is demonstrated for two single-nucleotide polymorphisms (118A > G, 17 C > T) of the *mu* opioid receptor gene (*OPRM1*). This study reiterates the importance of AIMs in defining ancestry, especially in admixed populations.

## Introduction

The well-established genetic differences between ancestral populations may have an effect on disease prevalence and outcomes, as well as on drug response [1]. The presence of subgroups that differ in allele frequencies is relevant to public health and has numerous clinical implications. Analysis of population structure using a clustering algorithm can distinguish between populations based on DNA polymorphisms [1]. Admixture occurs when a new hybrid population is formed from formerly isolated populations [2]. Estimating the proportions of different ancestries in admixed populations is especially important in case–control association studies, since spurious associations may occur due to population substructure [3,4]. To avoid spurious associations, researchers can adjust the regression model in an association study for individual admixture by estimating individual ancestry with a set of ancestry informative markers (AIMs) that have high allele frequency differences between continental groups.

African and Hispanic Americans constitute a large part of the US population (approximately 12% each). The majority of the African American (AA) population is a recently admixed population (average of approximately six generations, >200 years ago) generated primarily by forced migration from Africa and a diverse range of admixture with European Americans (EA) and Native Americans (NA).

The term Hispanics/Latinos refers to a diverse population of Latin American descent. The Hispanic/Latino complex genetic structure reflects over five centuries of historical confluence of three major parental populations: Native American, European (primarily from the Iberian Peninsula and Southern Europe), and West African populations, with very large variations in ancestry proportions that is a result of country-specific migration history. Several studies have reported the admixture of this population [e.g., [5-14]. The Hispanic subgroups in the USA were shown to differ in the prevalence of several diseases compared with African and European Americans. Admixture analysis may show whether genetics explains these differences and may allow discriminating between socio-demographic and genetic contribution.

This study was designed to determine the ancestry proportions of a large multi-ethnic sample, recruited for studies of drug addiction in the USA, with a set of 168 AIMs and to examine the concordance of self-reported ethnicities and family history with AIMs data.

## Materials and methods

### Sample

The sample of 1,291 subjects is part of a larger cohort that was recruited for genetic study of drug addiction in the Laboratory of the Biology of Addictive Diseases at The Rockefeller University, New York. This set included only subjects who self-reported to be AA, EA, or Hispanic American (HA), which are the major groups in our cohort. Subjects were recruited at The Rockefeller University Hospital, The Weill Medical College of Cornell University, New York, The Manhattan Campus of the VA NY Harbor Health Care System, and the Dr. Miriam and Sheldon G. Adelson Clinic for Drug Abuse Treatment and Research, Las Vegas. They included former opiate addicts in methadone maintenance treatment (*n* = 726), cocaine and/or alcohol addicts (*n* = 143), and healthy volunteers (*n* = 422). A subset of this sample (e.g., EA and AA opiate addicts and healthy volunteers) was included in our previously reported case–control association studies [15,16].

Subjects completed a three-generation family history questionnaire developed in our laboratory as part of their ascertainment interview. The family history questionnaire includes questions on (1) place of birth (i.e., city, state, country, and region); (2) self-identified race, ethnicity, or cultural group; and (3) nationality. Each question was answered for self, parents, grandparents, and great-grandparents, when known. The questionnaire was filled in by an experienced clinician interviewer. No pre-set list of categories was provided. Responses were assigned into nine categories: African, AA, EA, HA, Asian, Caribbean African, NA, Other/Mixed, and Unknown. Ambiguous responses were assigned by the authors based on geographical regions. All subjects signed an informed consent for genetic studies with a 99.7% consent rate [17]. The institutional review boards of The Rockefeller University, VA NY, and Cornell University approved the study. The Rockefeller University IRB also reviews the Adelson Clinic, Las Vegas.

### DNA preparation and genotyping

DNA was extracted from blood using the standard salting-out method. Two hundred and fifty to five hundred ng DNA was used for genotyping, as described [16]. Genotyping of AIMs and *OPRM1* single-nucleotide polymorphisms (SNPs) was performed on a 1,536-plex GoldenGate™ Custom Panel (Illumina, San Diego, CA, USA) [18]. Genotyping was performed at The Rockefeller University Genomics Resource Center according to the manufacturer's protocol (Illumina). Analysis was performed using BeadStudio genotyping software (Illumina). Genotype data were filtered based on SNP call rates (>99.5%) and cluster separation, and 18 AIMs were excluded from the analysis because of poor cluster separation (Additional file 1: Table S1). Random samples (approximately 10%) were genotyped in duplicate with high reproducibility rate (99.9%).

### Determination of individual ancestry by AIMs

The set of 186 unlinked AIMs was selected based on differences in allele frequency by at least 70% and 10-fold between at least two continental populations (from among European, African, and Asian populations of the HapMap project) (Additional file 1: Table S1) [19]. Genotypes of 168 AIMs with adequate quality were analyzed using STRUCTURE v.2.2 software [20] to obtain individual ancestry proportions (ancestry biographical score). Analysis was also performed for a known set of 1,051 subjects representing 51 worldwide populations (the Cell Line Panel of the Human Genome Diversity Project (HGDP)/Centre d’Etude du Polymorphisme Humain (CEPH)) [21].

The number of clusters (*K*) was defined by running the data with different *K* values and computing the probability of *K* = *n* in the HGDP sample. The seven-factor solution was optimal and closely replicated the seven-factor solution found for the same 51 reference populations that matched major geographical regions [1,22].

In the current analysis, the entire sample was analyzed simultaneously with the reference set in an ‘anchored’ approach that was shown to yield a stable factor structure interpretable in the context of worldwide genetic diversity [22-24]. An ancestry biographical score for seven clusters corresponding to geographical regions: Africa, Europe, Middle East, Central Asia, Far East Asia, Oceania (islands of the tropical Pacific Ocean), and America, was estimated for each individual, with reference to the 1,051 individual panel, and sum to 1 [22].

## Results

**Figure 1:**
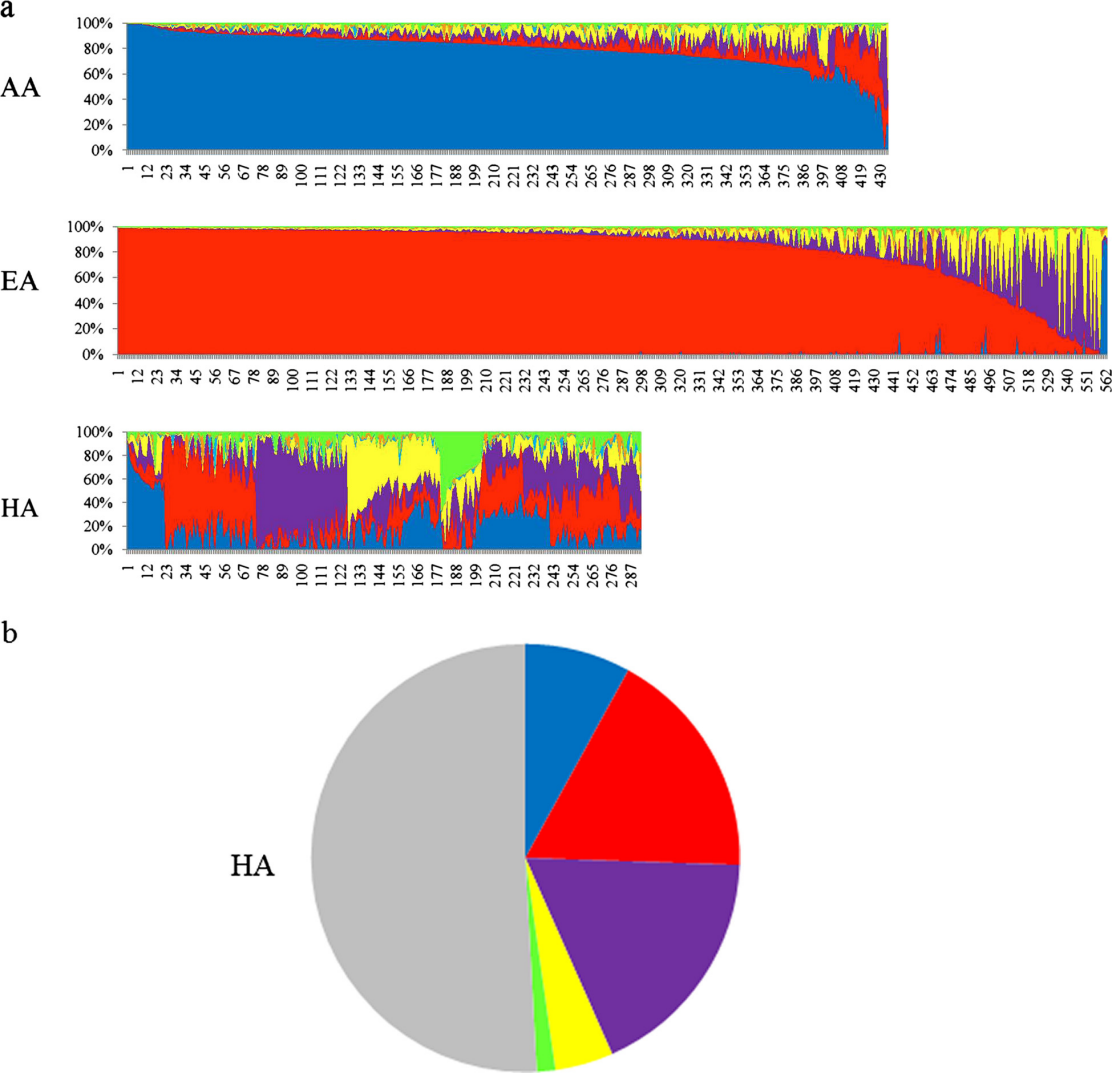
**Individual admixture estimates and the distribution of the major ancestry contributions in the HA group.** (**a**) Schematic representation of the individual admixture estimates using (*K* = 7). Each *vertical line* represents one individual, and subjects are displayed according to their predominant cluster contribution. The clusters correspond to the geographical regions based on the HGDP sample. Color code: Africa (blue), Europe (red), Middle East (purple), Central Asia (yellow), Far East Asia (cyan), Oceania (amber), and America (green). (**b**) The distribution of the major ancestry contributions (frequency > 0.5) in the HA group. Color code: no single major ancestry (gray); major ancestries: Europe (red), Middle East (purple), Africa (blue), Central Asia (yellow), and America (green).

A set of 1,291 subjects was selected for this study based on self-report ethnicity. This set is part of a larger cohort that was recruited for genetic study of drug addiction from New York City and Las Vegas. The subjects belong to the following self-reported groups: AA (*n* = 435), EA (*n* = 563), and HA (*n* = 293). Ancestry biographical scores for seven clusters (*K*) were obtained for each subject using STRUCTURE analysis of 168 AIMs. The analysis was performed for each sample along with 1,051 HGDP samples as a reference (see the ‘Materials and methods’ section). Based on the HGDP sample, the seven factors correspond to the geographical regions of Africa, Europe, Middle East, Central Asia, Far East Asia, Oceania, and America, and they are named by the geographical regions hereafter, for simplicity. Figure 1a shows the relative ancestry contributions for each subject in each of the three groups. There is a very small contribution of clusters corresponding to Oceania and Far East Asia in this sample.

### African Americans

Complete or almost complete (>10/14 possible answers) three-generation family history was available for 296 subjects (68%). Partial information was available for 87 subjects (20%), and no family data were available for 52 subjects including 3 adopted subjects (12%). Based on this data, the AA group can be divided into the following subgroups: AA for at least two generations with no report of another ancestry (41%), AA with some Caribbean ancestry, African Caribbean, AA with some Native American ancestry, AA with some European ancestry, mixed ancestry, new immigrants from Africa, and unknown ancestry (Table 1).

**Table 1:** Self-identification, family history, and ancestry biographical score based on AIMs

**Self-identified**	**Family history data**	***n***	**Clusters (*K*)**^**a**^				
			**1**	**2**	**3**	**4**	**5**
			**Africa**	**Europe**	**Middle East**	**Central Asia**	**America**
African Americans							
	African American	179	0.83	0.05	0.05	0.05	0.01
	Some Caribbean ancestry	32	0.79	0.07	0.05	0.05	0.01
	African Caribbean	40	0.82	0.05	0.05	0.05	<0.01
	Some Native American ancestry	59	0.79	0.07	0.06	0.05	0.02
	Some European ancestry	17	0.70	0.10	0.12	0.05	<0.01
	Mixed ancestry	48	0.72	0.06	0.08	0.06	<0.01
	New immigrants from Africa	8	0.94	0.01	0.02	0.01	<0.01
	No family data	52	0.74	0.09	0.07	0.08	<0.01
	*Total*	*435*	*0.80*	*0.07*	*0.06*	*0.05*	*0.01*
	(SD)		(0.12)	(0.08)	(0.07)	(0.05)	(0.01)
Hispanic Americans							
	Puerto Rican	181	0.18	0.27	0.30	0.14	0.07
	Other Caribbean or mixed Caribbean	35	0.35	0.27	0.20	0.11	0.04
	Latin American	22	0.20	0.20	0.19	0.17	0.19
	Caribbean/Latin American	6	0.09	0.27	0.31	0.16	0.13
	Puerto Rican with some European ancestry	15	0.19	0.33	0.26	0.14	0.05
	No data	34	0.17	0.30	0.24	0.17	0.09
	*Total*	*293*	*0.20*	*0.27*	*0.27*	*0.14*	*0.08*
	(SD)		(0.18)	(0.22)	(0.24)	(0.16)	(0.11)
European Americans							
	*Total*	*563*	*0.01*	*0.81*	*0.10*	*0.06*	*0.01*
	(SD)		(0.01)	(0.17)	(0.13)	(0.08)	(0.01)

^a^Data for the clusters corresponding to Oceania and Far East Asia are not presented (<0.02). *SD* standard deviation.

Based on AIMs analysis, the major ancestry contribution for the majority (96%) of the self-identified AA subjects is African, as is shown in Figure 1a. The second major contribution (0.26–0.40) is from Europe, Middle East, or Central Asia. The mean proportions of each of the major factors in the AA sample are shown in Table 1. The mean African contribution for the subgroups that were divided based on family history data was 0.80 for the subgroups of African Americans, African Caribbeans, some Caribbean ancestry, and some Native American ancestry. AIMs analysis detected a lower mean African contribution (0.70) in the sample that reported some European ancestry or mixed ancestry and a higher mean African contribution (0.94) in new immigrants from Africa, as expected, validating the sensitivity of this set of AIMs. Notably, there was a very small Native American contribution in all subgroups including those (*n* = 59) that reported some Native American ancestry.

Comparison between self-identification and AIMs data revealed 17 subjects for whom the contribution of the African factor was less than 50%. There were 13 subjects (3%) with an African contribution in the 0.25–0.5 range for which the other major contributing factor was European, Middle East, or Central Asia. There were four subjects (0.9%) with a <0.25 African contribution, but none of them had family history data.

### Hispanic Americans

Complete or almost complete three-generation family history was available for 202 subjects (71%). Partial information was available for 53 subjects (18%), and no family data were available for 32 subjects (11%) including 1 adopted subject. Based on the three-generation family history data, the self-identified HA subjects can be divided to the following subgroups: Puerto Ricans (62%), mixed or other Caribbean Islands, Latin Americans, Caribbean/Latin Americans, Puerto Ricans with European ancestry, and no data (Table 1).

Based on AIMs analysis, the distribution of individual admixture estimates in the HA sample show a wide range of ancestry proportions (Figure 1a). Of the 293 self-reported HA, 150 subjects (51%) have a hybrid pattern of five ancestries (all with frequency <0.5), 52 subjects (18%) have major European ancestry, 53 (18%) subjects have a major Middle Eastern contribution, 22 subjects (8%) have a major African ancestry contribution, 13 subjects (4%) have a major Central Asian contribution, and 3 subjects (1%) have a major Native American contribution (Figure 1b). The mean proportions of each of the five major clusters are listed in Table 1. It is clear that the reported family history does not reflect the complex ancestry contribution indicated by analysis of AIMs. Although the mean contributions from Far East Asia and Oceania were low (<0.02), 33 subjects have a >0.05 contribution from Oceania and 24 subjects show a >0.05 contribution from Far East Asia. There is a higher contribution of Native American in the Latin American subgroup (0.19) and the mixed Caribbean/Latin American subgroup (0.13) compared to other subgroups supporting the sensitivity of this set of AIMs.

### European Americans

AIMs analysis of the 563 self-reported European Americans showed that the major ancestry contribution was as follows (Figure 1a): 494 subjects (88%) have major European ancestry (>0.50), 33 (6%) subjects have a major Middle Eastern contribution, 12 (2%) subjects have a major Central Asian contribution, 4 (<1%) subjects have a major African contribution, 18 (3%) subjects have mixed contributions from Europe, Middle East, and Central Asia, and 2 subjects (<1%) show mixed contributions from Europe, Middle East, and Native America. The mean proportions of the major factors are shown in Table 1.

The comparison between family ancestry and AIMs in this group is beyond the scope of this study, as the specific AIMs were designed for continental populations and are of limited use in detecting substructure in closely related populations such as Europeans [19]. However, to identify potential conflicts between AIMs data and self-identified ethnicity, analysis of the family history data was performed for 34 subjects with a low European ancestry contribution indicated by AIMs analysis (<0.25). No family data were available for six subjects, including one adopted subject. As is shown in Table 2, out of the 18 subjects in which the major contribution indicated by AIMs analysis was from the Middle East, 6 subjects were Jewish (2 of them reported some non-European ancestry), 8 subjects were from Italy, Malta, Greece, or Iran, and 4 have no data available. We have recently showed a high contribution of the Middle East cluster in Israeli non-Ashkenazi Jewish subjects using the same set of AIMs [25]. The one subject, in whom a large Native American contribution was indicated by AIMs analysis, reported some Latin American ancestry. We found no support in the family history for the subjects in whom a major Central Asian (*n* = 9) or African contribution (*n* = 2) was indicated by AIMs.

**Table 2:** Family ancestry data in European Americans with <0.25 European ancestry

**Major ancestry (AIMs)**	***n***	**Family history**	***n***
Middle East	18	Jewish	6
		Italy, Malta, Greece	7
		Iran	1
		No data	4
Mixed Native American/Middle East	1	Latin America	1
Central Asia	10	Adopted	1
		Europe	7
		No data	2
Africa	4	Europe	2
		No data	2
Middle East/Central Asia	1	Europe	1
Total	34		34

### Allele frequencies of *mu* opioid receptor (*OPRM1*) SNPs in HA

To illustrate a situation of population-specific allele frequencies and their potential effect in admixed populations, allele and genotype frequencies were calculated for two missense polymorphisms (118A > G (rs1799971) and 17C > T (rs1799972)) in the *OPRM1* gene that plays an important role in opioid addiction [26]. Data from dbSNP, HapMap, and ALFRED show that the 118 G allele is common in Asians and Amerindians/Native Americans (0.35–0.5), is rare in African and Oceania populations, and occurs in moderate frequencies in European and Mexican populations (0.15–2.0). The 17C > T was not genotyped in the HapMap or ALFRED projects, but other studies and unpublished data from our laboratory indicate that it is African-specific (0.2–0.3) [27,28].

Analysis of genotype data from this study sample shows that the 17 T allele is rare in EA and HA (0.06) and its frequency in the AA group is 23%, in concordance with data from other studies (Table 3). The 118 G allele is rare in the AA group and has a frequency of 12% in the EA group and 17% in the HA group. AIMs data demonstrate that all the carriers of the 118 G allele have some European, Asian, and/or Native American contribution that could explain its origin. The frequencies of the 17 T and 118 G alleles and their related genotypes in the HA sample were significantly higher than those of European Americans (17% vs. 12%, and 6% vs. 2%, respectively, *p* < 0.004 for chi-squared test) (Table 3). This difference can be explained by the contribution of African and Asian ancestries. Since the contributions of different ancestries vary between different HA samples, the frequencies of the 118 G and 17 T alleles found in this sample may not represent the frequency in other HA samples.

**Table 3:** **Allele frequencies of*OPRM1*SNPs**

**Self-described**	**17 C > T (rs1799972)**		**118A > G (rs1799971)**	
	**C**	**T**	**A**	**G**
EA	0.98	0.02	0.88	0.12
AA	0.77	0.23	0.97	0.03
HA	0.94	0.06	0.82	0.17

EA vs. HA *p* < 0.004.

## Discussion

In this study, we have used a panel of 168 AIMs to estimate the ancestry composition of a multi-ethnic US sample collected for studies of drug addictions in New York City and Las Vegas. We compared this information to self-identified ethnicity and family history data. This comparison revealed high concordance in the major ancestry between self-identified ethnicity and AIMs analysis in African Americans and European Americans in agreement with other studies [10,29-31]. However, self-identified ethnicity and family history data could not predict the degree of admixture that may have an effect on allele frequencies.

This study reiterates the complexity of the ‘Hispanic/Latino’ term. Our results are compatible with studies indicating a relatively high European contribution (>50%) in subgroups of this population [5,7-9,12-14]. The study emphasizes the importance of AIMs data in genetic studies of HA since the self-identified ethnicity and family history may not reveal the complex ancestry contributions of this group. A special scrutiny has to be used in case–control association studies in this population, and AIMs data should be used to correct for potential population stratification.

The major Hispanic subgroup in this study was Puerto Rican, a population that currently represents approximately 1.5% of the US population. The pattern of ancestral proportions may have clinical significance for specific diseases when a specific ancestry may have a protective effect based on alleles with higher frequency in this population. For example, a recent study of end-stage kidney disease in Hispanics from New York City reported an approximately 30% African contribution and a very small Native American contribution [9] emphasizing the difference between ‘Mexican Hispanics’ and ‘Caribbean Hispanics.’ The sample in the current study was collected in Las Vegas and New York City and has a small, unrepresentative proportion of Hispanics of Mexican origin; conversely, studies with a mix HA from the East, the South West, and the West coasts of the USA are expected to have even larger level of admixture.

This study confirmed the finding of other studies showing a highly diverse proportion of European ancestry in self-identified AAs (7–21%) [9,22,23,30,32,33]. This diversity can be explained in part by the historical ‘one-drop rule’ (which classified individuals with any level of African ancestry as ‘African Americans’). It is clear that for the AA population, self-identified ethnicity is not sufficient to estimate the admixture level and a random AA sample may differ in admixture level from another sample, to an extent that will affect allele frequencies. The average of 7% of European admixture in this sample is compatible with some studies [22-24,33] but is lower than other studies [30,32,34,35]. This difference may be explained in part by the various numbers of defined clusters used in the different studies. Our STRUCTURE analysis was based on seven clusters, and the European cluster obtained in studies based on small number of clusters is most probably represented in our study by two clusters: Europe and Middle East. These clusters were found to be relatively close (population differentiation index *F*_st_ = 0.005) [36] and the Middle East cluster was shown to form a gradient across Europe [37]. Including the Middle East cluster in the total European contribution would result in a 13% contribution that is closer to the estimate by other studies. The difference between our estimates of ancestral proportions and other studies may also reflect recruitment from different US regions and the use of a different set of AIMs.

Our finding of a very low Native American contribution in this AA sample, based on AIMs analysis, is compatible with other studies [9,22,32] and may represent a conflict with some of the reported family history. It is also possible that this sample does not represent other AA groups in the USA. It most likely does not reflect a limitation in the AIMs set or the analysis since this cluster was clearly detected in HA.

In this study, we have shown the effect of admixture on allele frequencies of two SNPs in the *mu* opioid receptor gene, *OPRM1* (118A > G and 17C > T). The significantly higher frequency of the 118 G allele in this small random HA sample compared with the EA sample probably reflects the contribution of Asian and Native American ancestries. Similarly, the significantly higher frequency of the 17 T allele in the HA sample compared with the EA sample most probably reflects the contribution of African ancestry. This study reiterates the importance of AIMs in defining ancestry, especially in admixed populations and emphasizes the concept that Hispanic Americans is not a valid category in genetic research.

This study provides support for the robustness of this set of AIMs, as our results corroborate the results of other studies using this set [18,22-24]. The study demonstrates that computation of ethnic factor scores ‘anchored’ against worldwide genetic diversity (CEPH reference populations) yields a stable factor structure, allows comparisons between different datasets, and may permit combining data from different studies. This set of AIMs is especially useful in situations where large-scale genotyping is not available.

There are several limitations to this study: first, our sample does not represent the general US population, as it was derived from only two main locales (Las Vegas and New York City), with unrepresentative low proportion of Mexican Americans. Second, the specific AIMs used in this study were selected based on HapMap data (release #16c.1, 2005) [19] and as such are limited to allele frequency data from small samples of three main original HapMap populations (Northern and Western Europe (CEPH), Nigeria (Yoruba), and Han Chinese (Beijing)) and may not be suited for analysis of certain populations.

Albeit very promising, great care must be used in research of this kind to avoid misleading interpretations. Genetic ancestry estimates could help the dismissal of the concept of race, but may also support the notion of distinct human biological subgroups that may increase stigmatization and discrimination [38,39]. There is growing evidence that major health differences between populations involve gene-environment interaction, and, as such, their understanding will need not only genetic tools but also social/cultural information [22].

## Competing interests

The authors declare that they have no competing interests.

## Authors’ contributions

OL designed the study, carried out the genetic studies, performed data analysis and interpretation, and wrote and revised the manuscript. OA performed data analysis and interpretation. PHS performed STRUCTURE analysis. MA supervised subject recruitment, and ascertainment and acquisition of ancestry data of the LV sample. MJK supervised the overall subject recruitment, ascertainment and acquisition of ancestry data, and critically reviewed the manuscript. All authors read and approved the final manuscript.

## Supplementary Material

Additional file 1 **Table S1. ** List of AIMs. Click here for file
