# Supplementary material for: Estimating ancestral proportions in a multi-ethnic US sample: implications for studies of admixed populations
Source: Hum Genomics. 2012 Jul 5;6(1):2. doi: 10.1186/1479-7364-6-2 (PMC3437566; doi:10.1186/1479-7364-6-2)
Supplement: Additional file 1 — Table S1. List of AIMs. [file 1479-7364-6-2-S1.docx]

| **Supplementary Table 1. List of AIMs** | |
| --- | --- |
| SNPs | SNPs excluded |
| rs1001484 | rs1033372 |
| rs1002587 | rs10750836 |
| rs1003229 | rs10802184 |
| rs10107384 | rs1514626 |
| rs10113320 | rs1554817 |
| rs10152453 | rs1828774 |
| rs10152524 | rs2027760 |
| rs10249419 | rs2036565 |
| rs1028040 | rs2082380 |
| rs10420077 | rs2625956 |
| rs10488401 | rs267071 |
| rs10488619 | rs26880 |
| rs1052502 | rs4907376 |
| rs10745288 | rs6496858 |
| rs10763013 | rs6670693 |
| rs10785952 | rs679832 |
| rs10795588 | rs7158302 |
| rs10847171 | rs8110904 |
| rs10868793 |  |
| rs10879311 |  |
| rs10883533 |  |
| rs10884188 |  |
| rs10933921 |  |
| rs10935292 |  |
| rs11098964 |  |
| rs11126303 |  |
| rs11136793 |  |
| rs11184898 |  |
| rs11203006 |  |
| rs11713100 |  |
| rs1206920 |  |
| rs12125484 |  |
| rs1227647 |  |
| rs12313915 |  |
| rs12481662 |  |
| rs12595448 |  |
| rs12618959 |  |
| rs12644851 |  |
| rs12678324 |  |
| rs13134663 |  |
| rs1336978 |  |
| rs1347201 |  |
| rs1368928 |  |
| rs1375164 |  |
| rs1397529 |  |
| rs1402851 |  |
| rs1444893 |  |
| rs1446959 |  |
| rs1488299 |  |
| rs1494962 |  |
| rs1503079 |  |
| rs1507086 |  |
| rs1519260 |  |
| rs1532052 |  |
| rs1538956 |  |
| rs1552314 |  |
| rs1563382 |  |
| rs16877243 |  |
| rs1698042 |  |
| rs1716167 |  |
| rs1719982 |  |
| rs1811510 |  |
| rs1823778 |  |
| rs1827950 |  |
| rs1869237 |  |
| rs1871534 |  |
| rs1885167 |  |
| rs1894450 |  |
| rs1986420 |  |
| rs2004426 |  |
| rs2008592 |  |
| rs2039248 |  |
| rs2102727 |  |
| rs2193595 |  |
| rs2220128 |  |
| rs2220858 |  |
| rs2225979 |  |
| rs2231164 |  |
| rs2241083 |  |
| rs2345275 |  |
| rs2387137 |  |
| rs2388511 |  |
| rs2409710 |  |
| rs2416504 |  |
| rs2416791 |  |
| rs246760 |  |
| rs2486448 |  |
| rs2626018 |  |
| rs2730891 |  |
| rs2759281 |  |
| rs2823662 |  |
| rs2842063 |  |
| rs289816 |  |
| rs2927385 |  |
| rs2930125 |  |
| rs2934193 |  |
| rs2937067 |  |
| rs2948905 |  |
| rs3100865 |  |
| rs310644 |  |
| rs315280 |  |
| rs326626 |  |
| rs333113 |  |
| rs344816 |  |
| rs36110 |  |
| rs3786467 |  |
| rs3796285 |  |
| rs3814134 |  |
| rs3912537 |  |
| rs4240793 |  |
| rs4241398 |  |
| rs4265409 |  |
| rs4484738 |  |
| rs4511483 |  |
| rs4653130 |  |
| rs4687002 |  |
| rs4721415 |  |
| rs4737761 |  |
| rs4783432 |  |
| rs4787645 |  |
| rs4824001 |  |
| rs4841401 |  |
| rs4863731 |  |
| rs4889490 |  |
| rs4958667 |  |
| rs4968382 |  |
| rs4981115 |  |
| rs541805 |  |
| rs556399 |  |
| rs5753625 |  |
| rs590614 |  |
| rs6001728 |  |
| rs6023367 |  |
| rs6061779 |  |
| rs6074585 |  |
| rs6141319 |  |
| rs628825 |  |
| rs6414248 |  |
| rs6424922 |  |
| rs643272 |  |
| rs6472362 |  |
| rs650276 |  |
| rs6510332 |  |
| rs6552216 |  |
| rs655590 |  |
| rs6593430 |  |
| rs6595142 |  |
| rs6687440 |  |
| rs6718709 |  |
| rs6785846 |  |
| rs6790692 |  |
| rs6869589 |  |
| rs6926774 |  |
| rs692713 |  |
| rs6955490 |  |
| rs6991838 |  |
| rs711159 |  |
| rs7253691 |  |
| rs733370 |  |
| rs7397702 |  |
| rs7618370 |  |
| rs7692206 |  |
| rs7932809 |  |
| rs815608 |  |
| rs889548 |  |
| rs9318026 |  |
| rs9388989 |  |
| rs9639213 |  |
